# Sex chromosome evolution in parasitic nematodes of humans

**DOI:** 10.1038/s41467-020-15654-6

**Published:** 2020-04-23

**Authors:** Jeremy M. Foster, Alexandra Grote, John Mattick, Alan Tracey, Yu-Chih Tsai, Matthew Chung, James A. Cotton, Tyson A. Clark, Adam Geber, Nancy Holroyd, Jonas Korlach, Yichao Li, Silvia Libro, Sara Lustigman, Michelle L. Michalski, Michael Paulini, Matthew B. Rogers, Laura Teigen, Alan Twaddle, Lonnie Welch, Matthew Berriman, Julie C. Dunning Hotopp, Elodie Ghedin

**Affiliations:** 1 Division of Protein Expression & Modification, New England Biolabs, Ipswich, MA 01938 USA; 2 Department of Biology, Center for Genomics and Systems Biology, New York University, New York, NY 10003 USA; 3 Institute for Genome Science, University of Maryland School of Medicine, Baltimore, MD 21201 USA; 4 Wellcome Sanger Institute, Wellcome Genome Campus, Hinxton, CB10 1SA UK; 5 Pacific Biosciences, Menlo Park, CA 94025 USA; 6 School of Electrical Engineering and Computer Science, Ohio University, Athens, OH 45701 USA; 7 Laboratory of Molecular Parasitology, Lindsley F. Kimball Research Institute, New York Blood Center, New York, NY 10065 USA; 8 Department of Biology and Microbiology, University of Wisconsin Oshkosh, Oshkosh, WI 54901 USA; 9 European Molecular Biology Laboratory, European Bioinformatics Institute, Wellcome Genome Campus, Hinxton, Cambridge, CB10 1SD UK; 10 Department of Surgery, UPMC Children’s Hospital of Pittsburgh, Pittsburgh, PA 15224 USA; 11 Department of Microbiology and Immunology, University of Maryland School of Medicine, Baltimore, MD 21201 USA; 12 Greenebaum Cancer Center, University of Maryland School of Medicine, Baltimore, MD 21201 USA; 13 Department of Epidemiology, School of Global Public Health, New York University, New York, NY 10003 USA

**Keywords:** Evolutionary genetics, Eukaryote, Genome, Genome evolution, Parasite genomics

## Abstract

Sex determination mechanisms often differ even between related species yet the evolution of sex chromosomes remains poorly understood in all but a few model organisms. Some nematodes such as *Caenorhabditis elegans* have an XO sex determination system while others, such as the filarial parasite *Brugia malayi*, have an XY mechanism. We present a complete *B. malayi* genome assembly and define Nigon elements shared with *C. elegans*, which we then map to the genomes of other filarial species and more distantly related nematodes. We find a remarkable plasticity in sex chromosome evolution with several distinct cases of neo-X and neo-Y formation, X-added regions, and conversion of autosomes to sex chromosomes from which we propose a model of chromosome evolution across different nematode clades. The phylum Nematoda offers a new and innovative system for gaining a deeper understanding of sex chromosome evolution.

## Abstract (web summary)

Many nematode worms, including *Caenorhabditis elegans* have XX/XO sex determination, while other species have XY. The authors use a new genome assembly of the filarial parasite *Brugia malayi* and published data to show that nematode sex chromosome evolution is highly plastic.

## Introduction

Nematodes are the most abundant animals on earth^1^. At least a third of the human population is infected with a nematode at any given time^2^. Phylogenetic analyses have resolved nematode species into five broad clades with parasitic representatives in each^3,4^. Nematodes can be free-living, like *Caenorhabditis elegans* (Clade V), which is used as a model organism in genetic studies, or parasitic, like *Brugia malayi* (Clade III), the most intensely studied human filarial nematode. *B. malayi* can be maintained in the laboratory using feline, rodent, and insect hosts, and its genome was the first reported for any parasitic nematode^5,6^. Eight species of filariae infect humans causing significant morbidity, disability, and socioeconomic loss in developing regions of the world^7^.

From an evolutionary perspective, the filariae represent an interesting contrast to the model nematode *C. elegans*, which has, like many other nematodes, XX/XO sex determinism, while filarial species can have Y chromosomes^8–15^. It is generally held that evolution of heteromorphic sex chromosomes (e.g. X and Y) begins when a pair of homologous autosomes acquires a sex-determining factor^16^. Sex-specific genes are typically linked to this factor and recombination becomes suppressed in the heterozygous sex to facilitate inheritance of these genes en bloc. This suppression of recombination between the proto-sex chromosomes can be mediated by various mechanisms and typically expands to the majority of the Y chromosome resulting in heteromorphic chromosomes, as the Y chromosome degenerates following gene decay through mutation^16–20^. This necessitates some form of dosage compensation to account for the 2:1 ratio of sex-linked genes in the homogametic sex relative to the heterogametic sex^16,17,19,20^. Nature has evolved numerous variations to this generally accepted theory of sex chromosome evolution and sex determination. For example, neo-sex chromosomes can also evolve in situations where a sex chromosome pair already exists, either by fusion to an autosome or by acquisition of a new sex-determining factor on an autosome, both leading to sex chromosome turnover^16^.

The absence of a Y chromosome in many studied nematodes suggests the XO sex chromosome system is the ancestral state in nematodes, while XY is a derived state^11,21^. This led to the suggestion that the Y chromosome evolved once in the ancestor of all filarial nematodes^11^. Using our new assembly of the complete *B. malayi* chromosomes, and chromosome information from other parasitic and non-parasitic nematodes, we explore the evolution of nematode sex chromosomes. In contrast to the prevailing model, our comparative genome analyses reveal a dynamic evolutionary path in filarial nematodes involving multiple neo-Y and neo-X chromosomes.

## Results and discussion

### Genome assembly and tracing of chromosome evolution

Using single molecule sequencing (PacBio), optical mapping, and manual curation, we assembled the *B. malayi* genome into five chromosomes, with only eight gaps (Supplementary Table 1). With an N50 of 14.2 Mb, this improves substantially on the previous assemblies^6^ and is one of very few parasitic nematodes for which essentially complete chromosome assemblies are available^22^. The assembly process also led to a closed mitochondrial genome and a closed *Wolbachia* genome—the symbiotic partner of a number of filarial worms. Over 97% of the 248 CEGMA (Core Eukaryotic Genes Mapping Approach) genes were identified; four absent genes (corresponding to KOG IDs KOG1468, KOG2303, KOG2531, and KOG2770) were found to be missing in all filarial genomes, and one gene (corresponding to KOG1185) found in the current *B. malayi* assembly, is absent in other filariae^22^. No methylation was detected in the PacBio sequencing. The optical maps resolved five telomeres, two on each of chromosomes 2 and 4, and one on chromosome 3. Consistent with earlier karyotyping, centromeres were not identified on any of the chromosomes, supporting the hypothesis that, like *C. elegans*^23^, filarial nematodes have holocentric chromosomes^24^.

**Fig. 1:**
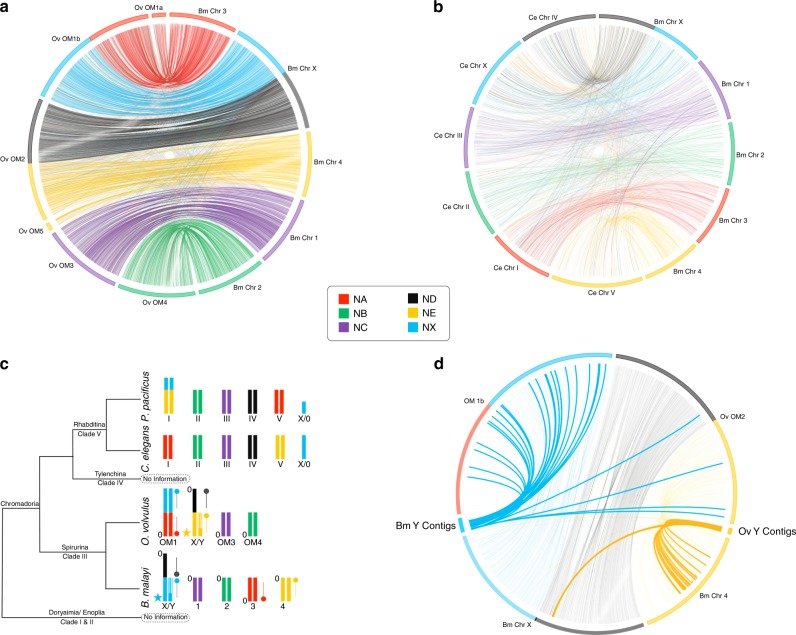
Homology between *B. malayi*, *O. volvulus*, and *C. elegans* chromosomes using conserved blocks. The *B. malayi* chromosomes were mapped against the **a** *O. volvulus* and **b** *C. elegans* chromosomes. The correlation between chromosomes was predicted using comparisons of chromosome homology between *B. malayi, O. volvulus*, and *C. elegans*. The nematode chromosomes were broken into Nigon elements, with the coloring based on corresponding regions in *C. elegans* chromosomes. **c** A model of chromosome evolution is provided according to clades^3,4^, where each Nigon element is color-coded and described by the number of the corresponding *C. elegans* chromosome. The stars denote the PAR in both *O. volvulus* and *B. malayi*, color-coded by element, and the pins appearing next to the chromosomes denote the orientation of the Nigon elements between these two species, with “0” representing where the contig in the fasta file begins relative to the diagram. The portion of the Y chromosome that is significantly diverged from the X chromosome yet maintains identity with the Nigon element is illustrated with stripes. The Y chromosomes between *O. volvulus* and *B. malayi* have arisen twice from two different autosomes, while the X chromosomes also show significantly different composition, which further correlates to the composition of the Y chromosome. Despite these differences, in both *B*. *malayi* and *O. volvulus*, ND is now part of the X chromosome and is *1N*. **d** Putative  *B. malayi* Y contigs are mapped to relevant *O. volvulus* chromosomes, and putative *O. volvulus* Y contigs are mapped to relevant *B. malayi* chromosomes. The conserved sequences between the *B. malayi* chromosome Y-specific contigs and the *O. volvulus* NX span 150 kbp, while they span only 15 kbp for ND and 7–9 kbp for all the remaining Nigon elements. When combined with the PAR, this suggests that chromosome Y is largely derived from NX.

While intrachromosomal rearrangements are common in nematodes, and obliterate local synteny within chromosomes, interchromosomal rearrangements are rare and most genes maintain an association with a given chromosome over long evolutionary periods^6,25–28^, even between diverse taxa like *C. elegans* (Rhabditina) and *Trichinella spiralis* (Enoplia)^28^. To examine chromosome evolution, we used Nigon elements, previously defined ancestral linkage groups analogous to *Drosophila* Muller elements^29,30^. We assigned Nigon homology by pairwise PROMER^31^ alignments between chromosomes of *B. malayi*, the related filarial parasite *O. volvulus*, and *C. elegans* (Fig. 1a, b, and Supplementary Fig. 1). This helped us develop a model of chromosome evolution for the filaria and other nematode species across several clades (Fig. 1c).

Sequencing depth differences between males and virgin females (Supplementary Fig. 2a, b) reveal that the largest *B. malayi* chromosome (24.9 Mb) is the X chromosome (Table 1). To determine which regions of the genome were 1*N* or 2*N*, we calculated the number of copies per cell (*N*) of chromosomes, Nigons, and contigs by dividing the mean sequencing depth of each region by half the mean sequencing depth for the genome. While most of the X chromosome in the male worms is 1*N*, there is an ~2.6 Mb putative pseudoautosomal region (PAR) starting at 22.3 Mb with 2*N* sequencing depth that is shared between the X and the Y chromosomes (Supplementary Fig. 2c, d). Consistent with this, males display many heterozygous single nucleotide variants (SNVs) in this putative PAR region (Supplementary Fig. 2e, f).

**Table 1:** Chromosome characteristics.

Chromosome	Size (bp)	% GC	Gaps (bp)	Predicted *N*
X	24,943,668	29.4	12,977	1.19 ± 0.03
1	14,701,151	28.1	110,166	2.04 ± 0.04
2	14,214,749	28.0	0	2.04 ± 0.03
3	13,951,302	27.9	152,426	2.05 ± 0.04
4	13,467,244	27.8	1796	2.00 ± 0.04
Mitochondria	13,657	24.5	0	90.85 ± 25.65
*Wolbachia*	1,080,084	34.2	0	9.08 ± 4.24

Based on karyotyping data^14^, the Y chromosome of *B. malayi* is expected to be similar in size to the autosomes, but it was not fully recovered in the assembly. Using the sequencing depth of males and virgin females, 64 contigs (Supplementary Data 1) were identified as putative chromosome Y-specific with >0.8*N* depth in males and very little sequencing depth in virgin females (Supplementary Fig. 3 and Supplementary Data 1). These include a contig (Bm_024) containing a significant match to the Y chromosome marker, *tag on Y* (TOY)^15^. Most of these contigs correspond to Nigon-X (NX), suggesting that the X chromosome of *C. elegans* and the Y chromosome of *B. malayi* share a common ancestry. Many of the contigs are predicted to have a high copy number (Supplementary Data 1), indicating that they may contain collapsed repeats, making assembly difficult for the Y chromosome.

Given the deduced copy number of the putative chromosome Y-specific contigs and the ~2.6-Mb estimate of the PAR region, the identified putative Y chromosome contigs span 17.3 Mb, which represents 70% of the size of the X chromosome, a size that is consistent with the karyotyping. While the assembled size of the genome is ~81 Mbp (Table 1), this suggests the complete *B. malayi* genome is likely ~96 Mbp, also consistent with previous estimates^14^.

The *Onchocerca volvulus* PAR (OvPAR) and Y-specific contigs^22^ are different from those of *B. malayi* (Fig. 1d). While the predicted Y contigs of *B. malayi* are largely from NX and map to *O. volvulus* chromosome 1, those of *O. volvulus* are from Nigon-E (NE) and map to *B. malayi* chromosome 4 (Fig. 1d). There is in fact only a single gene (Bm17149)—encoding a protein with an aspartic protease domain and containing a CCHC-type zinc finger—that is conserved on the Y-specific contigs of both *B. malayi* and *O. volvulus*.

*B. malayi*, like other members of the Onchocercidae family, was expected to have four autosomes, with both X and Y chromosomes (4A + XY)^11^. Because *O. volvulus* and *O. gibsoni*, also in the Onchocercidae, are 3A + XY, it led to the hypothesis that the 3A + XY state was derived from the 4A + XY, which assumes a single 4A + XY state. However, while the *B. malayi* X chromosome is a fusion of Nigon-D (ND) and NX, the *O. volvulus* X chromosome (OM2) is a fusion of ND and NE (Fig. 1a). Remarkably, the sex chromosome of *C. elegans* is not part of the *O. volvulus* X chromosome. These observations indicate that there are clearly multiple 4A + XY states in filarial nematodes.

**Fig. 2:**
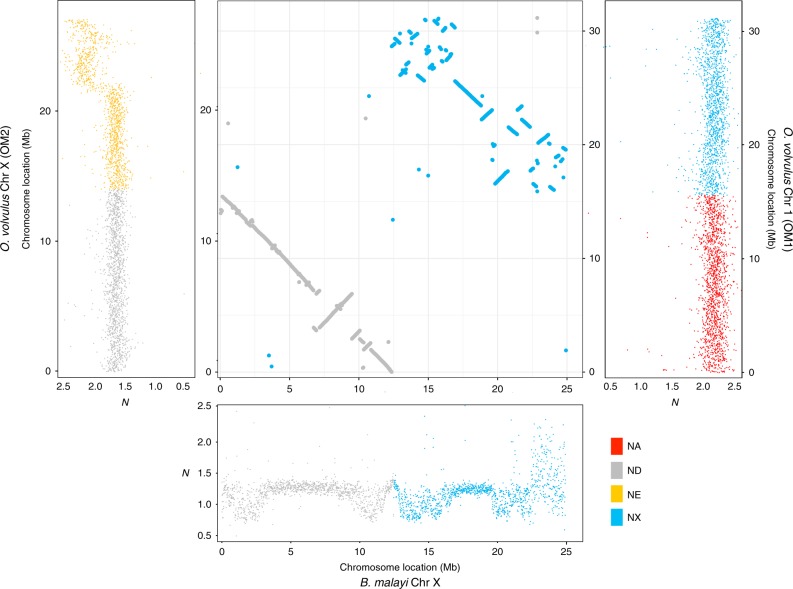
*B. malayi* Chr X mapping across *O. volvulus* Chr 1 (NX) and Chr X (ND). An analysis of the *B. malayi* X chromosome indicates that it is the product of a fusion of two Nigon elements: ND and NX, in contrast with the *O. volvulus* X chromosome, which is composed of a fusion between ND and NE. The middle panel, which includes combined mummerplots to highlight Nigon element matches, shows the synteny of the *B. malayi* X chromosome to its corresponding two Nigon elements in *O. volvulus*. Each axis is marked with a corresponding sequencing depth plot of that chromosome. Thus, the *X*-axis of the main plot is linked to the sequencing depth of the *Brugia malayi* X chromosome, which is divided into ND (gray) and NX (blue). The left *Y*-axis shows the corresponding *O. volvulus* chromosome X, composed of ND (gray) and NE (yellow), while the right *Y*-axis shows the corresponding *O. volvulus* chromosome 1, composed of NA (red) and NX (blue). In both *B. malayi* and *O. volvulus*, ND is fused to a Nigon element that was likely autosomal in the most recent common ancestor, but those fusions occur at opposite ends of ND in the two nematodes.

**Fig. 3:**
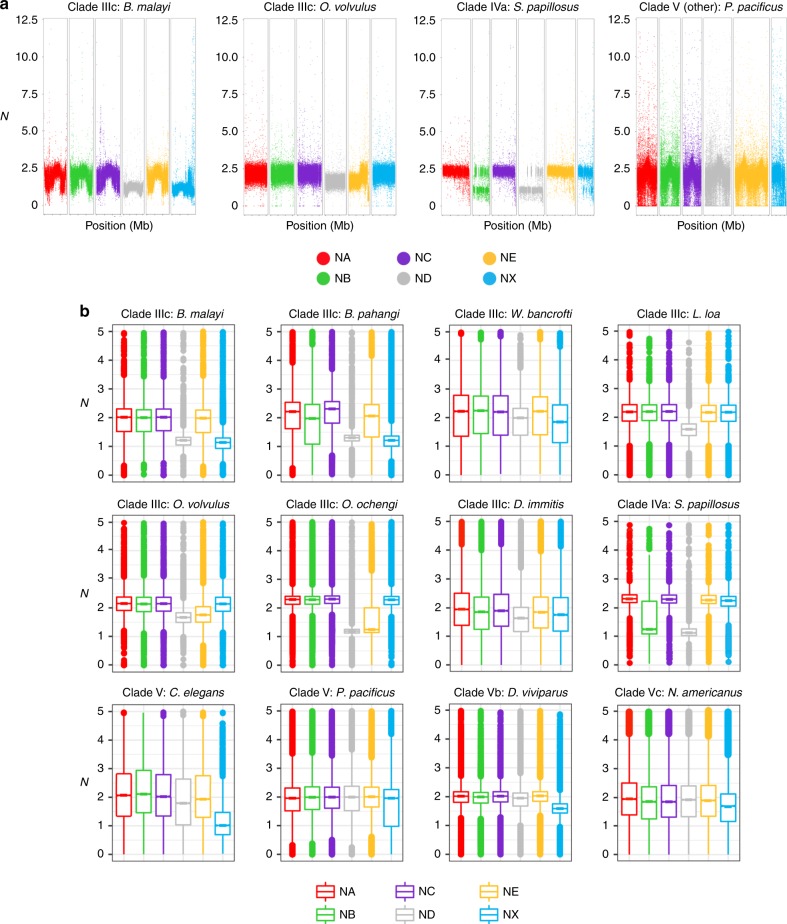
Sequencing depth per Nigon element for diverse nematodes. We provide examples of species across clade III, IV, and V nematodes. **a** The calculated *N* (average sequencing depth over a 10 kb window normalized to half the average sequencing depth of the whole diploid genome) is visualized for each of the Nigon elements for a representative set of species in different clades. Haploid regions are likely contiguous, but not visualized as such in these plots since data is plotted by the contig order in the reference genome. On the *X-*axis, distances between ticks represent 5 Mb. **b** Box plots showing the calculated *N* according to Nigon element reveals that *B. malayi*, *B. pahangi*, and *W. bancrofti* all share similar unpaired chromosome segments in males corresponding to ND and NX. *L. loa* appears to only be unpaired in males for ND, while *O. volvulus* and *O. ochengi* are unpaired in males for ND and NE. *D. viviparus* and *N. americanus* are unpaired in males for only NX, while *P. pacificus* is only partially unpaired in males for NX, as expected and previously described. The center line of the box plots is drawn at the median of the depth at each Nigon element, the upper and lower hinges are at the 25% and 75% quartiles of the depth, while the whiskers extend 1.5× the interquartile range. Outliers are plotted as points outside of that range.

In both *O. volvulus*^22^ and *B. malayi*, the ND portion of the X chromosome is unpaired in males but the fusion to the NE or NX, respectively, occurs at opposite ends of ND (Figs. 1c and  2). To determine whether ND is unpaired in males of other filarial nematodes, we compared the sequencing depth of contigs across Nigon elements using publicly available sequence data (Supplementary Data 2) for additional filarial species: *B. pahangi, Wuchereria bancrofti,* *Loa loa*, *O. ochengi, O. flexuosa*, and *Dirofilaria immitis* (Fig. 3 and Supplementary Fig. 4). The majority of ND appears to be universally unpaired in male filarial nematodes (Fig. 3). In contrast, NX is at least partially unpaired in males for all clade V nematodes (Rhabditina) including *Pristionchus pacificus*, *Dictyocaulus viviparus*, and *Necator americanus* relative to *C. elegans* (Fig. 3 and Supplementary Fig. 4). The *P. pacificus* X chromosome was previously shown to be derived from part of NX with the remainder of NX as a NX/NE fused autosome^32^. Analysis of available sequence data for *Strongyloides papillosus*, a clade IV nematode, reveals that ND is haploid (Fig. 3), and may have fused to a sizable portion of Nigon-B (NB) (Fig. 3). These results are consistent with prior results showing chromosomal diminution due to a fusion between an autosome (NB) and a chromosome that is homologous to the X chromosome in sister taxa^33^, which we predict are NB and ND, respectively. This suggests that ND is the ancestral X chromosome in at least clade III, IV, and V, which we expect to be monophyletic within Nematoda, and that a neo-X chromosome has evolved from an autosome in the ancestor of clade V nematodes including *C. elegans* with a corresponding conversion of a sex chromosome to an autosome. Neo-X chromosomes arose at least twice more through an X-added region (XAR) in filarial nematodes with the NX/ND fusion in *Brugia* and *Wuchereria* and the ND/NE fusion in *Onchocerca*. The previously described fusion in *P. pacificus*^32^ resulted in a neo-X chromosome in this clade V species. In addition, neo-Y chromosomes appear to have evolved from different autosomes at least twice in filarial nematodes, likely after the evolution of the neo-X chromosome via a XAR. Intriguingly, a neo-X/neo-Y system with an ND/NX fusion has also been observed in *C. elegans*^34^. In this system, it is NX that is unpaired in males, and not ND, as observed in *B. malayi*.

Although there are no common Nigon elements between the X chromosomes of *B. malayi*, *O. volvulus*, and *C. elegans*, and interchromosomal rearrangements are uncommon in nematodes^6,25–28^, 278 genes are shared on the X chromosomes of all three organisms despite the widespread lack of conservation of linkage groups. Among these genes we observed an overrepresentation of functional terms for G-protein-coupled receptors (GPCRs), fibronectin type III proteins, and immunoglobulins (Supplementary Data 3). Given the evolutionary distances, it is difficult to discern any smaller scale rearrangements to understand the movement of these genes—whether through chromosomal fusion, gene translocations, or retrotransposition. Further sequencing aimed at generating complete chromosomes from more filarial and non-filarial nematodes is needed to fully resolve this issue.

We also determined whether any conserved dosage compensation genes could be identified in *B. malayi*. Using BLASTp we found homologs to four *C. elegans* genes involved in dosage compensation, *sdc-1*, *dpy-21*, *dpy-27*, and *dpy-30*. While the first three are found on the *B. malayi* X chromosome, the homolog to *dpy-30* is located on chromosome 4. Other genes involved in dosage compensation in *C. elegans*—including *sdc-2*, *sdc-3*, *dpy-26*, *dpy-28*, and *xol-1*—did not have homologs in *B. malayi*.

### Enrichment of repeat elements on sex chromosomes

Since sex chromosomes frequently accumulate repetitive DNA^35,36^, we compared the content of various repeats on the sex chromosomes with those on autosomes. *B. malayi* has two very well-characterized repetitive DNA elements, the HhaI and MboI repeats. HhaI is in tandemly arrayed copies of a ~322 bp monomer and has been successfully used as a real-time PCR target for detection of *B. malayi* infections from patient blood^37,38^. MboI is less conserved, with monomers that show considerable variation in sequence and length^39^. The HhaI tandem DNA repeats were previously thought to constitute close to 12% of the genome^40^, but the initial assembly of the genome^6^ as well as our current assembly only identifies HhaI as 1.6% of the genome, with a read-based analysis of sequencing data from 22 males suggesting 2.6 ± 1.6%. The HhaI repeats are over-represented on the small haplotype contigs as well as the unplaced contigs not attributed to chromosome Y or haplotypes (Fisher’s Exact Test, *p*-value < 0.00001) (Supplementary Data 4, Supplementary Fig. 5a). The HhaI repeats tend to occur at the ends of the chromosomes consistent with being subtelomeric repeats (Supplementary Fig. 5a).

In contrast, MboI repeats were overrepresented on the putative chromosome Y contigs (Fisher’s Exact Test, *p*-value < 0.00001) (Supplementary Data 4, Supplementary Fig. 5b) with 21% of the MboI sequence on these contigs being attributed to BmMbo8^39^, considered the prototypic member of this family of repeats. In fact, 90% of the BmMbo8 sequences were found specifically on the putative chromosome Y contigs, while the remaining 10% are located on one end of NB as well as the small, unplaced contigs not attributed to chromosome Y or haplotypes.

PAO-type LTR retrotransposons, another well-characterized repeat, are enriched on the putative Y contigs. They are also enriched on ND of the *B. malayi* X chromosome compared to the autosomes (Fisher’s Exact Test, *p*-value < 0.00001) (Supplementary Data 4, Supplementary Fig. 5a), but not on NX. Many of the retrotransposons present on the X chromosome (30%) are flanked by sequence resembling the SL1 spliced leader (SL) elements (Supplementary Fig. 5a). SLs in nematodes are 22 nt leader sequences that are normally trans-spliced onto the 5′-end of transcripts from 100 nt SL RNAs^41^. In *C. elegans*, 70% of mRNAs have a trans-spliced leader sequence (either SL1 or SL2); in *B. malayi*, the SL is identical to that of *C. elegans* SL1. From our annotation of *B. malayi* genes, we estimate that 70% (8300/12,000) of genes have SL1 addition sites. We found 316 elements in the genome containing an SL1 signature. Of these, 196 (62.0%) were a close match to the 22 nt region of the 100 nt SL1 RNA gene. The remaining 120 SL1 represent integral SL1 sequences within the *B. malayi* genome, not associated with a 5S rRNA or an array of SL1 RNA-encoding genes. These sequences are scattered across the chromosomes with 60 of these within 1 kb of PAO retrotransposons (Supplementary Data 5). An occasional configuration was two SL1 sequences flanking a PAO-type LTR retrotransposon with both SL1 sequences on the same strand. This pattern of SL1 integration indicates that at some point spliced leaders were added onto expressed PAO retrotransposons and integrated with them into the genome.

Another class of multi-copy *B. malayi* repeat that could contribute to chromosome remodeling are the lateral gene transfers (LGT) from *Wolbachia*^42^. *B. malayi*, like a number of filarial nematodes, carry obligate intracellular bacterial *Wolbachia* endosymbionts that frequently transfer their DNA to their host, creating nuwts (for nuclear *Wolbachia* transfers). In this assembly there are 345 nuwts spanning 428,883 bp (Supplementary Fig. 5, Supplementary Data 6). These correspond to portions of 133 unique *Wolbachia* protein coding genes of which 59 are present more than once with 5 having >10 copies, confirming that nuwts are novel repeat families in the *B. malayi* genome (Supplementary Data 6). Surprisingly, nuwts are significantly under-represented on NX (Fisher’s Exact Test, *p*-value = 0.00022) (Supplementary Fig. 5, Supplementary Data 4), while they are over-represented on small unplaced contigs not attributed to either chromosome Y or haplotypes (Fisher’s Exact Test, *p*-value < 0.00001) (Supplementary Fig. 5, Supplementary Data 4). The vast majority of nuwts are pseudogenized protein-coding regions with frameshifts, large insertions/deletions, and premature stop codons. Twenty-one full-length protein-coding regions can be found in the nuwts although some have altered start and stop codons when compared to the *Wolbachia* coding sequences (Supplementary Data 6).

### Sex-biased gene expression

In *C. elegans*, genes with high female-biased expression (i.e. up-regulated in females as compared to males) are enriched on the X chromosome and genes with high male-biased expression are depleted^43^, but genes that show low levels of sex biased expression do not follow this pattern^43^. To determine whether there were genes on the *B. malayi* X chromosome with sex-biased transcription, we used publicly available transcriptome data of *B. malayi* stages during development to adulthood^44^. We identified a significant number of *B. malayi* genes that displayed sex-biased gene expression in males and females at 30 and 120 days post infection (dpi) (Supplementary Data 7). It should be noted that at 30 dpi, all male worms have reached the L4 stage while females may represent a mix of molting L3 and L4 as they usually complete their molt by day 34^45^. The most sex-biased expression was found to be at 120 dpi, with 2858 genes (24%) exhibiting male-biased expression, and 2666 genes (23%) exhibiting female-biased expression. However, females at 120 dpi are gravid, and therefore the observed gene expression may be germ-line enriched rather than sex-biased.

**Fig. 4:**
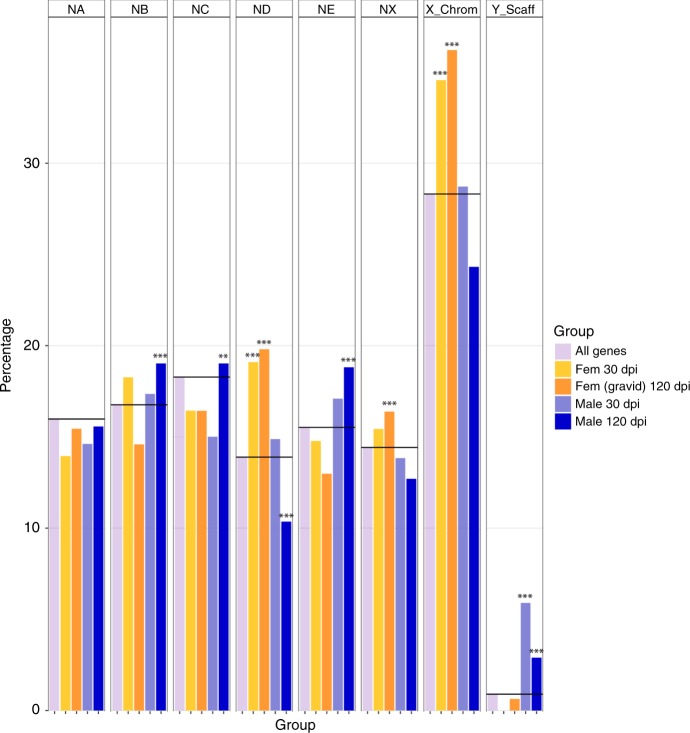
Sex-biased gene enrichment. The percentage of male and female sex-biased genes at 30 and 120 dpi are shown relative to each Nigon element as well as the X chromosome and the Y-specific contigs. The percentage of sex-biased genes is compared to total genes on each particular Nigon element, chromosome, or set of contigs, which is shown as the black line. The stars represent significant statistical enrichment or depletion of sex-biased genes on that particular element as determined by Fisher’s exact test.

Nigon elements and the Y-specific contigs were analyzed to determine if sex-biased genes were enriched or depleted on any one specific element. At both 30 and 120 dpi, the X chromosome of *B. malayi* was significantly enriched for female-biased genes, equally on the regions that correspond to ND and NX (Fig. 4). Male-biased genes were enriched on Y-specific contigs at both time points, as well as on autosomal NB, NC, and NE at 120 dpi, while being under-represented on ND at 120 dpi. We are not aware of any prior report of sex-biased gene expression on an autosome. The BmPAR was also enriched for male-biased genes. An examination of the expression of only genes on the Y-specific contigs reveals a number of genes with increased expression in the adult males at both 30 and 120 dpi (Fig. 4).

Given the presence of sex-biased motifs in the *C. elegans* genome, we searched for enriched motifs in potential transcription factor-binding sites (TFBSs) in the promoter regions of sex-biased genes. Our search for sex-biased motifs yielded 13 enriched motifs at 30 dpi (Supplementary Data 8) and 16 at 120 dpi (Supplementary Data 9). DME_ATCAATTAA is among the TFBSs enriched in females at 30 dpi, with homology to the known motif M1223_1.02, which is the predicted binding site of the transcription factor Vab-3 in *C. elegans*. In *C. elegans*, Vab-3 is involved in anterior/posterior patterning, regulation of cell adhesion, male tail tip morphogenesis, and cell fate commitment^46^. Three TFBSs enriched at 30 dpi in females—Improbizer_TTCTAACCTAATTAATT, Homer_10_1, and DME_GACCYADW—have homology to the known motif M4937_1.02 which is the predicted binding site of nhr-85, a nuclear hormone receptor involved in the development of the egg-laying system and formation of SDS-resistant dauer larvae^47^.

### Sex chromosome evolution is informed by nematode genomics

Heteromorphic sex chromosome evolution begins with a sex-determining factor followed by the emergence of recombination suppressing features, such as inversions, that prevent movement of sex-determining factors between a homologous pair of chromosomes^16,17,20,48^. However, a small portion—the PAR—continues recombining and aids in chromosome segregation as well as pairing during meiosis^17,20^. Between *C. elegans, B. malayi*, and *O. volvulus*, intrachromosomal rearrangements occur less frequently on chromosome X (Fig. 2) than on autosomes (Supplementary Fig. 6), which suggests that chromosome X recombination is arrested. With the arrest of recombination, other portions (i.e. non-PAR regions) of the Y chromosome are predicted to become highly heterochromatic with profound gene loss from Muller’s ratchet, background selection, and genetic hitchhiking^16,17,20,48^. Our results are consistent with this hypothesis, with many likely heterochromatic repeats like Mbo8, being enriched on Y-specific contigs (Supplementary Fig. 5b).

Natural selection is thought to favor the evolution of reduced recombination between a sex-determining gene and nearby genes with sex-specific effects^16,17,20,48^. Unless recombination is already arrested^48^, recombination appears to cease in stages on the Y-chromosome such that strata develop over time, with the youngest strata closest to the PAR. In a naturally occurring neo-X/neo-Y NX/ND fusion in *C. elegans*, crossovers were found to occur in the most distal region to the fusion^49^. That is consistent with the results here where the PARs are distal to the fusion. However, when sex is determined by sex chromosome dosage (e.g. XX/XO systems), it is less clear how this recombination arrest might occur, and as such strata may not be present^48^. Nematodes are important taxa for studying this aspect of sex chromosome evolution since XX/XO systems are common. Yet the lack of nematode Y chromosome sequences has stymied such an investigation. Gene amplification has been observed with Y-specific genes, which may be an adaptive trait enabling Y-to-Y gene conversion^48^. For example, OVOC12909 (which encodes a protein of unknown function, DUF1759), the only shared Y-specific gene in both *O. volvulus* and *B. malayi* (Supplementary Data 10), is present in multiple copies on putative Y contigs in *B. malayi*.

### Summary

The results presented here point to a system of sex determination in *B. malayi* and other filarial nematodes that differs substantially from the model nematode, *C. elegans*. Heteromorphic sex chromosome evolution seems to have occurred numerous times in the filarial nematodes creating natural replicate experiments in sex chromosome evolution. Future research should focus on resolving this further including complete sequencing of the two neo-Y chromosomes. Further sequencing is also needed to elucidate how widespread PARs are in nematodes. Small PARs may be missing in karyotyping data and not apparent with fragmented genome assemblies. Recent advances in ultra-long sequencing (e.g. Oxford Nanopore sequencing) are likely to enable such future studies. Future work using a comparative approach will shed more light on sex chromosome evolution and sex determination in nematodes and give us a unique opportunity to observe the evolution of sex chromosomes and the diversity of sex determination systems.

## Methods

### Parasite material

All *B. malayi* parasite material was obtained from intraperitoneal infections of gerbils (*Meriones unguiculatus*) maintained by TRS Labs (Athens, GA, USA) or by the FR3 (Filariasis Research Reagent Resource Center; BEI Resources, Manassas, VA, USA). The FR3 and TRS life cycles have been maintained independently for decades, but were initiated with material from the same clinical infection^50^. Live adult parasites were shipped from TRS Labs to New England Biolabs (NEB) for preparation of DNA for sequencing on the Pacific Biosciences single molecule real-time sequencing platform and for optical mapping.

Infected gerbils were shipped from the FR3 to the University of Wisconsin, Oshkosh for recovery of virgin female worms. The care, maintenance, and treatment of the animals used in this study followed protocols approved by the University Institutional Animal Care and Use protocol (#0026-000246-R1-09-14). Sexually immature L4 and adult female worms were collected from gerbils that were euthanized 24–27 dpi. Worms were collected by flushing the peritoneal cavity with 37 °C RPMI-1640 (ThermoFisher, Waltham, MA, USA) supplemented with 0.4 U/mL penicillin + 0.4 µg/mL streptomycin (Sigma-Aldrich, St. Louis, MO, USA) and 2 µg/mL heparin (Sigma-Aldrich, St. Louis, MO, USA). The sex of worms was determined by light microscopy, then the worms were washed in 1x PBS and flash frozen. All females were determined to be unmated based on the absence of sperm at the junction of the oviduct and the ovary.

### Purification of *B. malayi* DNA and RNA for sequencing

For sequencing on the Pacific Biosciences platform, high molecular weight *B. malayi* genomic DNA was prepared by grinding frozen worms in liquid nitrogen and transferring the ground material to 100 mM Tris–HCl (pH 8.5), 50 mM NaCl, 50 mM EDTA, 1% (v/v) SDS, 1.1% (v/v) β-mercaptoethanol. Proteinase K (NEB, Ipswich, MA, USA) was added to 100 µg/ml and the sample rocked gently at 55 °C for 4 h. DNA was recovered by phenol–chloroform extraction with gentle phase mixing by rotation on a shaker rotisserie (ThermoFisher, Waltham, MA, USA) followed by centrifugation at 4000 × *g*. DNA was spooled from solution following ethanol precipitation of the aqueous phase in the presence of 0.2 M NaCl and transferred to TE (pH 8.0). RNase A (Epicentre, Madison, WI, USA) was added to 25 µg/ml and the sample incubated at 37 °C for 1 h. DNA was re-extracted with phenol–chloroform and precipitated once more by centrifugation at 12,000 × *g* at 4 °C. DNA size and concentration were assessed using gel electrophoresis and a Nanodrop spectrophotometer (ThermoFisher, Waltham, MA, USA). DNA was extracted from virgin female worms by first disrupting tissues using the TissueLyser LT (Qiagen, Germantown, MD, USA), following the protocol for purification of DNA from plant tissues. DNA was then purified using the QIAamp DNA Micro Kit (Qiagen), following the isolation of genomic DNA from tissues protocol. β-mercaptoethanol (11.1 ng/ml) was added to the Proteinase K incubation step to help disrupt nematode cuticle. Final DNA concentration was determined via NanoDrop spectrophotometry.

For the SL1 analysis, total RNA was purified using the TRizol Plus Kit (Invitrogen). Frozen *B. malayi* microfilaria, L3, L4, adult male, or adult female worms were removed from dry ice and ground with a disposable plastic pestle and 0.1 mm silica spheres in Trizol (Invitrogen). The worms were returned to dry ice, and then the grinding process repeated three more times. Chloroform was added, the tube shaken by hand and placed at RT for 2 min. To separate phases, the tube was spun at 12,000 × *g* for 15 min at 4 °C. The clear aqueous phase containing the RNA was removed to a new tube and ethanol added to a final concentration of 35%, followed by hand mixing for 30 s. The RNA was then purified using a spin cartridge and centrifuged at 12,000 × *g* for 15 s. Total RNA was eluted with three sequential spins of 75 µl RNase-free water. Polyadenylated mRNA from each life stage was purified from total RNA with the Dynabeads mRNA purification kit (Invitrogen) following the manufacturer’s protocol.

### DNA and RNA sequencing

For PacBio sequencing, 30 µg genomic DNA was randomly sheared to 20 kb using a G-tube device and the manufacturer’s recommended procedure (Covaris, Inc., Woburn, MA). The SMRTbell template library was prepared using the standard protocol. The final sequencing library was size-selected using the Blue Pippin (Sage Science, Inc., Beverly, MA, USA) high-pass protocol with a 7 kb size cut-off. The library was sequenced using 24 SMRT cells on the RSII instrument with P5C3 chemistry, and 180 min sequencing time. Genomic DNA from 76 *B. malayi* virgin females (27 dpi, L4) was used to prepare 500 bp fragment size, amplification-free IIlumina libraries^51^. The library was run on an Illumina HiSeq v4, generating 75 bp paired-end reads.

For SL1 sequencing, stage-specific mRNA was barcoded and converted into cDNA using Superscript III Reverse Transcriptase (Invitrogen). Second strand synthesis was performed, targeting only the transcripts containing an SL1-leader by adding 2 µl 10X Thermopol Buffer (NEB), 1 µl 10 µM dNTP mix, 0.2 µl 50 µM biotin-labeled SL1-primer, 0.4 µl NEB Taq polymerase, and 5.4 µl H_2_O to the RNase-treated cDNA. The second strand reaction was purified from excess unused Biotin-SL1 primers with a Qiagen MinElute kit, and eluted into a total of 40 µl. Finally, Dynabeads M-280 Streptavidin beads were used to purify the SL1 containing dscDNA by following the manufacturer’s instructions, allowing for 20 min of binding and re-suspension in low TE buffer. PCR was performed on each barcoded life stage with Accuprime Taq Hifi (Invitrogen). 4N stretches were added at the beginning of both amplification primers to help cluster calling on the HiSeq. No template and 4N-DMX only PCR reactions were performed to insure amplification was occurring only from SL1-containing transcripts. PCR amplifications were gel purified in the range of 200–400 bp using the Qiagen MinElute kit following the manufacturer’s instructions. Due to low yields additional PCR reactions were performed on each life stage as above but with the size-selected template as the input, a reduction in the number of cycles to 15, and no final extension. All PCRs were combined on ice and purified on a MinElute column to create a final sequencing pool. Final sample pools were sent to Axeq Technologies (Korea). Pools were adapter ligated to produce a final library and ran on a single lane of the HiSeq 2000.

### Bioinformatic methods for SL1 identification

Reads from SL1 purified libraries were demultiplexed using an in-house script, then searched for SL1 signature sequences using nucmer^52^ in order to identify the read pair containing the SL1 sequence, and exclude potential contaminating sequences. To simplify downstream analysis, reads containing the SL1 sequence were subsequently treated as the forward read (i.e. if detected on reverse read, the read pairs were swapped). Reads were then split into separate libraries by barcode and each was aligned against the *B. malayi* genome (Bm.v4.QC.fa) using SMALT v0.5.2 (https://www.sanger.ac.uk/science/tools/smalt-0). The resulting SAM file was filtered on the bit string and CIGAR string to ensure that the first base of the forward read aligned. If the first base failed to align, this read was discarded. Read counts for each site were predicted individually for each stage-specific library, and then pooled into a single table. Predicted splice sites were associated with genes by constructing a sql database of the Bm.v4.QC.fa annotation, then querying the predicted site coordinates against the coordinates for the full gene length, including a 400 bp region upstream of the gene. Sites were then classified as either internal or external splice sites depending on whether they fell within the leader or body of the gene. Comparison of site usage was performed using multiple pairwise comparisons between L3, L4, adult male, adult female, and microfilarial stages using multiple comparisons with edgeR.

### Optical mapping

*B. malayi* male worms were washed in PBS then placed individually into disposable plug molds (Bio-Rad, Hercules, CA, USA). Approximately 50 µl of 1% (w/v) Incert Agarose (Lonza, Rockland, ME, USA) in PBS, held at 50 °C, was added and the plugs solidified at 4 °C for 1 h. Plugs were extruded into 1 ml of 1% (w/v) N-lauroylsarcosine, 2 mg/ml Proteinase K in 0.5 M EDTA (pH 9.5) held at 50 °C and then incubated overnight on a rocking platform in a 50 °C oven. The plugs were then washed five times for 1 h each wash in TE (pH 8.0) on a rocking platform at 4 °C and stored at 4 °C in 0.5 M EDTA (pH 8.0). For optical mapping, DNA molecules were stretched and immobilized along microfluidic channels before digestion with the restriction endonucleases *Spe*I and *Afl*II (NEB, Ipswich, MA, USA), yielding a set of restriction fragments ordered by position along the genome. The fragments were fluorescently stained and visualized to determine the fragment sizes. Assembling overlapping fragment patterns of single molecule restriction maps produced an optical map of the genome. The *B. malayi Spe*I optical map (created from an assembly of molecules >550 kb) consists of 17 contigs, an assembled size of 96.58 Mb and ~80× genome coverage of optical data. The *B. malayi Afl*II optical map (created from an assembly of molecules >575 kb) consists of 12 contigs, an assembled size of 77.57 Mb and ~80× genome coverage of optical data. The optical data were generated and analyzed using the Argus Optical Mapping System from OpGen (Gaithersburg, MD, USA) and associated MapManager and MapSolver software tools. Additionally, OpGen’s GenomeBuilder software was used to generate optical map assemblies from the sequence contigs to provide additional mapping information.

### *B. malayi* genome assembly and improvement

An unpublished *B. malayi* assembly in Wormbase WS242 (9827 contigs with an assembled size of 94,136,248 bp and a contig N50 of 191,089 bp) was generated from a mixture of capillary^6^, 454 (3, 8, and 20 kb mate pair libraries) and Illumina (500 bp paired end and 3 kb mate pair libraries) sequence data^42^. In this study a total of 11.3 Gb of long read data produced on the PacBio RSII instrument passed the 0.75 quality filter. A long-read de novo assembly was produced using HGAP 2.0^53^ and consisted of 1371 contigs with an assembled size of 90,313,157 bp and a contig N50 of 160,895 bp. The Pacific Biosciences assembly contained the *Wolbachia* genome whereas this had been removed from the WS242 assembly.

The PacBio de novo assembly was compared to the de novo *Spe*I optical map using MapSolver (OpGen, Gaithersburg, MD, USA). The lack of gaps in the PacBio assembly enabled many short contigs to be aligned against the optical contigs, resulting in fewer map gaps than WS242. The alignments were confirmed and further refined using the *Afl*II optical map. A gap5 database ‘hybrid assembly’ was created by mapping the component reads (capillary, 454 and Illumina) of the WS242 assembly to the PacBio contigs. The additional paired-end data this provided combined with the long-range optical mapping data provided evidence for scaffolding the assembly in gap5^54^, a genome visualization and editing tool. Sequences were manually grafted into the PacBio assembly using gap5 when sequence data from WS242 mapped to sequences either side of a scaffold gap in the hybrid assembly (from inspection of sequences aligning to the optical contigs in MapSolver). Once this process was complete the hybrid assembly contained the best sequence data from the two input assemblies. Three iterations of sequence correction were undertaken using ICORN2^55^ using Bowtie v.2.2.3 mapping with fake reads taken from the WS242 assembly contigs, followed by an additional three iterations using Illumina reads. Automated gapfilling (24 iterations) was performed using IMAGE^56^. PBJelly^57^ and Quiver (https://www.pacb.com/support/software-downloads/) were used to further close gaps, add additional scaffolding (PBJelly), and error correct and trim (Quiver) with PacBio data. Finally, ICORN was run once more (three iterations) to correct any errors introduced by the Quiver process and the sequence scaffolds were checked back against the optical map. Corrected PacBio reads were created from the PacBio-filtered subreads using Sprai-0.9.9.1 (http://zombie.cb.k.u-tokyo.ac.jp/sprai/index.html) and aligned back to the hybrid assembly to provide additional evidence for manual extension of sequence contigs. This allowed further placement of sequence contigs into scaffolds and gap-closure within scaffolds. Following this process, the total gap count in the assembly was reduced to 8. The completeness of the assembly was assessed by CEGMA v2 analysis^58^, to report the percentage of full or partial gene orthologs of 248 highly conserved eukaryotic gene families.

### Nigon element determination in *B. malayi*, *O. volvulus*, and *C. elegans*

To visualize the chromosomal rearrangements between *B. malayi* and other nematodes with well-assembled genomes, the new *B. malayi* v4.0 genome was compared against the *O. volvulus* and *C. elegans* genomes using the Promer tool from the MUMmer alignment suite^31,52^. Hits with <80% sequence homology between any two genomes were discarded, and the resulting alignments between the major chromosomes were plotted using R, Circos^59^, and mummerplot. Nigon elements were assigned based on *C. elegans* chromosomes: chromosome I was assigned to Nigon-A, chromosome II to NB, chromosome III to Nigon-C, chromosome IV to ND, chromosome V to Nigon-E, and chromosome X to NX. While the original description of Nigon elements included a Nigon-N^30^, we find no evidence for a seven-element ancestor. Therefore, we opted for a six-element nomenclature that seems to reflect the ancestral state of Spirurina, Tylenchina, and Rhabditina nematodes and possibly all members of Chromodoria. We opted for using NX over NN to preserve the link to the *C. elegans* chromosome nomenclature.

### Nigon element determination in other nematode genomes

In order to determine and visualize conserved sex chromosomal elements in nematode genomes beyond *B. malayi*, *O. volvulus,* and *C. elegans*, the most current genome assembly for each species was compared to *B. malayi*, *O. volvulus*, and *C. elegans* using the Promer tool from the MUMmer alignment suite^31,52^. Each contig of every nematode genome included in this analysis was assigned to a Nigon element based on chromosome Promer matches to *B. malayi*, *O. volvulus*, and *C. elegans*, based on Nigon terminology defined in Tandonnet et al.^30^. The Nigon element that covered the largest fraction of the contig when all three Promer matches were combined was assigned to that contig.

### Generation of data/images for Nigon elements

Contigs of the same Nigon element were concatenated together in the same order that they are listed in their respective FASTA files in order to analyze the sequencing depth of the element as a whole. In the case of *Onchocerca volvulus* and *B. malayi*, where the genomes are complete and there are chromosomal fusions, this involved breaking the fused chromosomes into the respective pieces. In some cases (*S. ratti*, *T. muris*, *T. spiralis*), there was a similar reduced chromosome number as a result of fusions but they could not be easily broken due to a large number of intrachromosomal rearrangements following fusion. This leads to an underrepresentation of Nigon elements due to the assignment of fused chromosomes to only a single Nigon element (see Supplementary Fig. 4). Illumina HiSeq or MiSeq data (depending on species) was downloaded (Supplementary Data 2) and mapped to each nematode genome using BWA MEM (v. 0.7.12), and the resulting BAM file was sorted and had its duplicates removed using Picard Tools (v. 2.5.0). The depth was calculated using SAMTOOLS depth (v. 1.3.1), with settings to include all bases (-aa) and not limit depth (max. depth = 10^8^). The contigs in each of these depth files was converted to Nigon elements based on the contig to element assignments created by the Promer analysis of that genome, and the resulting Nigon depth files were visualized in R. The R package ggplots (v. 3.1.0) was used to visualize the depth per 10 kb across each Nigon element.

Box plots of the depth in each Nigon element per 10 kb were generated with geom_boxplot in ggplots with the default parameters. The center line is drawn at the median of the depth at each Nigon element, the upper and lower hinges are at the 25% and 75% quartiles of the depth, while the whiskers extend 1.5× the interquartile range. Outliers are plotted as points outside of that range.

The major mode of the sequencing depth of each Nigon element for each species was calculated with the density function in the core R package using data where *N* > 0.2. The major modes of each Nigon element and the density distributions were used to determine putative haploid regions that likely correspond to sex chromosome-associated elements in each genome relative to the diploid autosomal Nigon elements. In addition to plots, a table was constructed of every nematode analyzed this way, which includes sample characteristics where available and the sex determination system of each nematode species.

### Identification of Y chromosome contigs and putative PAR

Illumina paired end reads from a pool of virgin females and 22 individual males (see Supplementary Data 1 for accession numbers of individual worm data) were mapped to the *B. malayi* genome with BWA MEM (bma 0.7.12) and positional sequencing depth was calculated using the DEPTH function of SAMtools v1.1^60^. The *Wolbachia* genome was included in the mapping but reads primarily mapping to it were excluded from further analysis. Average sequencing depth of the genome, as well as each contig, was calculated based on the sum of the sequencing depth at all positions divided by the total number of bases per contig. Copy number for each contig was then calculated from the ratio of contig sequencing depth to genomic sequencing depth.

Putative chromosome Y contigs were defined as contigs that were >0.8*N* in males and <0.7*N* depth in virgin females and had a male/female sequencing depth ratio of >4. The PAR was identified by examining the ratio of sequencing depth in the males to the virgin females across 100 kb windows along chromosome X. A putative PAR was identified as the large contiguous region of the X chromosome where no bins were defined as female dominated (ratio < 0.8). The cumulative length of the Y-specific contigs was calculated to be the sum of the calculated copy number of each predicted Y-chromosome contig multiplied by the contig length. The calculated DNA totals were added to the ~2.6 Mb PAR to provide an estimate of the size of chromosome Y.

### Determination of sex-biased gene expression and enrichment

Publicly available transcriptomic data^44^ from worms from L4 stage through to adulthood were mapped to the new *B. malayi* v4.0 genome. Genes were determined as sex-biased using edgeR (v3.26.8)^61^. Significantly differentially expressed genes were determined between male and female worms at 30 and 120 dpi using an FDR cutoff of 5%. Enrichment on chromosomes or Nigon elements was determined using Fisher’s exact test with a Bonferroni correction.

### Identification of *Wolbachia–Brugia* LGT

Putative LGT from *Wolbachia* sp. *w*Bm to *B. malayi*, termed nuclear *Wolbachia* transfers (nuwts) were identified by manually curating an aggregate of nucmer v.3.23 and blastn and blastx searches in blastall v.2.2.26 against the *w*Bm genome and predicted proteins [https://www.ncbi.nlm.nih.gov/nuccore/NC_006833.1]. Because nuclear mitochondrial transfers (numts) often interfere with the proper prediction of nuwts, numts were also predicted using the *B. malayi* mitochondrial genome. NUCMER from the MUMMER package was used with MAXMATCH. NCBI BLASTN and BLASTX searches were performed with an *e*-value of 1e–15. Once an initial set of nuwts were predicted they were excised from the genome and searched against the predicted *w*Bm (NC_006833) using PRAZE [http://ber.sourceforge.net/]. Frameshifts, truncations, and premature stop codons were manually counted from the PRAZE results. Curation, including specific BLAST-based searches of NT/NR, resulted in refining nuwt boundaries, eliminating some nuwts that were merely conserved between bacteria and eukaryotes, and reclassifying putative nuwts as LGT from other bacterial origins.

### Identification of SL1 genes, intrinsic SL1s, and PAO retrotransposons

SL1 sequences within the genome were identified using Nucmer, and the query sequence “GGTTTAATTACCCAAGTTTGAG”. PAO retrotransposons were identified using Exonerate 2.2.0 (https://www.ebi.ac.uk/about/vertebrate-genomics/software/exonerate) and multiple queries of peptide sequences of published PAO retrotransposon sequences 170596945, 170593441, 170592491, 170589333, 170589149, 170588831. rRNA sequences were identified using Infernal 1.1rc1^62^. SL1 sequences were classified as either occurring within standard SL1 gene arrays, or as intrinsic sequences elsewhere in the genome, either flanking PAO retrotransposons, or isolated. For an SL1 sequence to be considered as flanking a PAO retrotransposon, it had to occur within 1000 bp of either the predicted start or end of the PAO retrotransposon. To examine the homology of SL1-PAO elements, for each SL1 sequence 5′ to a PAO element the downstream 1000 bp were retrieved, and similarly for SL1 sequences 3′ to a PAO element, the upstream 1000 bp were retrieved. Two alignments were generated from these using Muscle (v3.8.31)^63^, and phylogenetic trees for each were generated using PhyML(v 3.1)^64^ with the GTR substitution model, and rates across sites modeled on an alpha distribution approximated using four site rate categories.

### Promoter motif discovery

Motif discovery was performed on the promoters of up-regulated genes and randomly selected background genes. There were two sets of differentially expressed genes, namely male vs. female at 30 and 120 dpi. For each set, there were two lists of up-regulated genes, namely the up-regulated genes in male worms and the up-regulated genes in female worms. Since the motif discovery process can be computationally inefficient on large gene sets, a small set of top up-regulated genes (i.e., foreground genes) was selected at different cutoffs (Supplementary Table 2). Promoter sequences were retrieved from the WormBase ParaSite Biomart web interface, capturing 1000 bp upstream of the translation start site for each gene. Background promoters were randomly selected from *B. malayi* genes, excluding the up-regulated genes. The background size was three times larger than the foreground.

### Ensemble motif discovery

Four motif discovery tools were used: GimmeMotifs 0.10.0b6^65^, DME 2.0^66^, DECOD v1.01^67^, and gkm-SVM 1.3. GimmeMotifs^65^ is an ensemble of generative motif discovery (i.e., no real background sequences needed) tools, including HOMER 2.0^68^, AMD 1.0^69^, BioProspector 1.0^70^, MDmodule 1.0^71^, MEME 4.11.2^72^, Weeder 2.0^73^, GADEM v1.3^74^, and Improbizer 1.0^75^. The parameters were: *motif_size* = large; *fraction* = 0.7. DME^66^, DECOD^67^, and gkm-SVM are discriminative motif discovery tools. Parameters for DME were: *motif_size* = 8,10,12,13,14,15,16,17; *motif_number* = 300. Parameters for DECOD were: *motif_size* = 8,10,12,14,16,18,20,22; *motif_number* = 10; *number_of_iteration* = 20. Parameters for gkm-SVM were: *motif_size* = 8,10,12,14,16,18,20,22; *kmer_size* = 10; *maxMismatch* = 2; *informative_columns* = 8; *alpha* = 15.0; *top_frac* = 2; *nMaxPWM* = 10.

To select statistically significant motifs, the motifs were assessed by a random forest classifier using scikit-learn^76^. Both Gini impurity^77^ and information gain^78^ criteria were used to evaluate the motifs. The union of the top 40 motifs that resulted from applying each criterion was retained. A Z-test was used to evaluate the significance of motif enrichment. The observed value was the frequency of a motif in the up-regulated genes. The expected value and standard deviation were calculated based on bootstrap sampling from the background promoters. The significance level (*p*-value) was 10^−3^.

A collection of 163 known nematode transcription factor-binding sites (TFBSs) were retrieved from MEME suite (http://meme.sdsc.edu), searching the motif databases JASPAR CORE 2016 nematodes^79^, CIS-BP *B. malayi*^80^, and uniprobe worm^81^. The remaining motifs were matched to known TFBSs using TOMTOM 4.11.2^82^. The motif similarity *p*-value threshold was 10^−4^.

Conservation analysis was performed using an adaptation of a published method^83^. Orthologous information between *B*. *malayi*, *C*. *elegans*, and *O*. *volvulus* was retrieved from Wormbase ParaSite Biomart^84^. Promoter regions of 1000 bp upstream from the translation start sites were extracted and CLUSTALW2^85^ was used to perform multiple sequence alignment with a gap open penalty of 10 and extension penalty of 0.1. A motif was defined as conserved if it occurred at the same position in the orthologous promoter region alignment of either *C. elegans* or *O. volvulus*.

### Reporting summary

Further information on research design is available in the Nature Research Reporting Summary linked to this article.

## Supplementary information

Supplementary Information

reporting summary

description of Additional Supplementary Files

Supplementary Dataset 1

Supplementary Dataset 2

Supplementary Dataset 3

Supplementary Dataset 4

Supplementary Dataset 5

Supplementary Dataset 6

Supplementary Dataset 7

Supplementary Dataset 8

Supplementary Dataset 9

Supplementary Dataset 10

## Data Availability

*B. malayi* v4 assembly with the WS270 annotation are available in the European Nucleotide Archive (ENA) database under accession number GCA_000002995.5, as well as at WormBase (http://www.wormbase.org/species/b_malayi) and WormBase-Para-Site (http://parasite.wormbase.org/Brugia_malayi_prjna10729/Info/Index/). Illumina HiSeq 2000 paired-end sequencing data from virgin female resequencing data are available at ERS992391. The PacBio data is available under PRJNA421950. Spliced leader RNAseq reads are available under PRJNA525735. Accession numbers for each dataset obtained from public data, and used in the analyses, are listed in Supplementary Data 2.
