# Supplementary material for: Sex chromosome evolution in parasitic nematodes of humans
Source: Nat Commun. 2020 Apr 23;11:1964. doi: 10.1038/s41467-020-15654-6 (PMC7181701; doi:10.1038/s41467-020-15654-6)
Supplement: Supplementary file 1 — Supplementary Information [file 41467_2020_15654_MOESM1_ESM.pdf]

Supplementary Information

Sex chromosome evolution in parasitic nematodes of humans

Foster, Grote, Mattick et al.

This PDF file includes:

Supplementary Figures 1-6

Supplementary Table 1

Supplementary Table 2

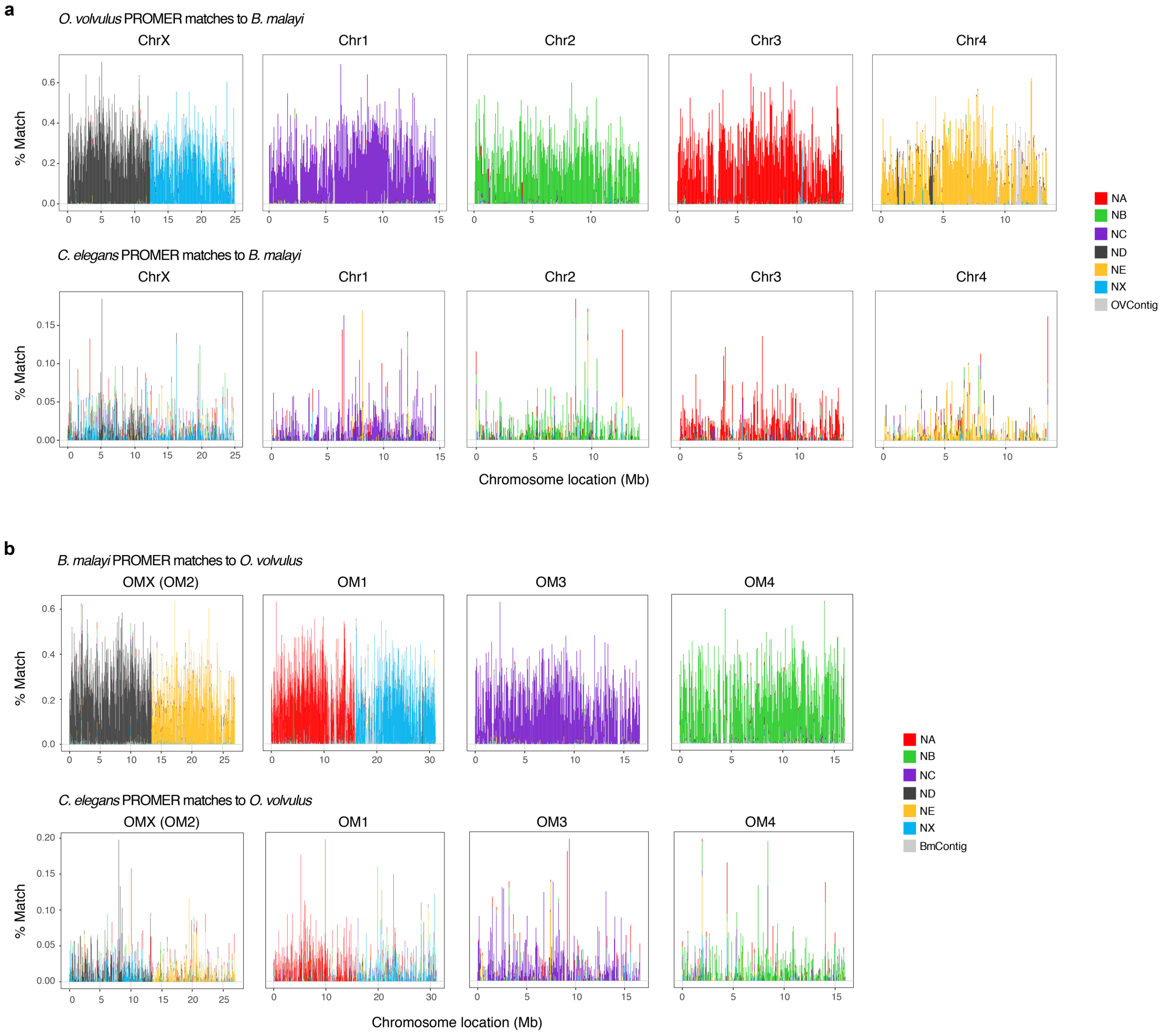

Supplementary Figure 1. Nigon element assignment (NA to NX) across the *B. malayi* and *O. volvulus* genomes

The percentage of a 10 kb window with a promer match (% match) was identified and color-coded by Nigon element. **(a)** Matches between *O. volvulus* and *B. malayi* (top) and between *C. elegans* and *B. malayi* (bottom) are visualized across the *B. malayi* genome. **(b)** Matches between *B. malayi* and *O. volvulus* (top) and between *C. elegans* and *O. volvulus* (bottom) are visualized across the *O. volvulus* genome.

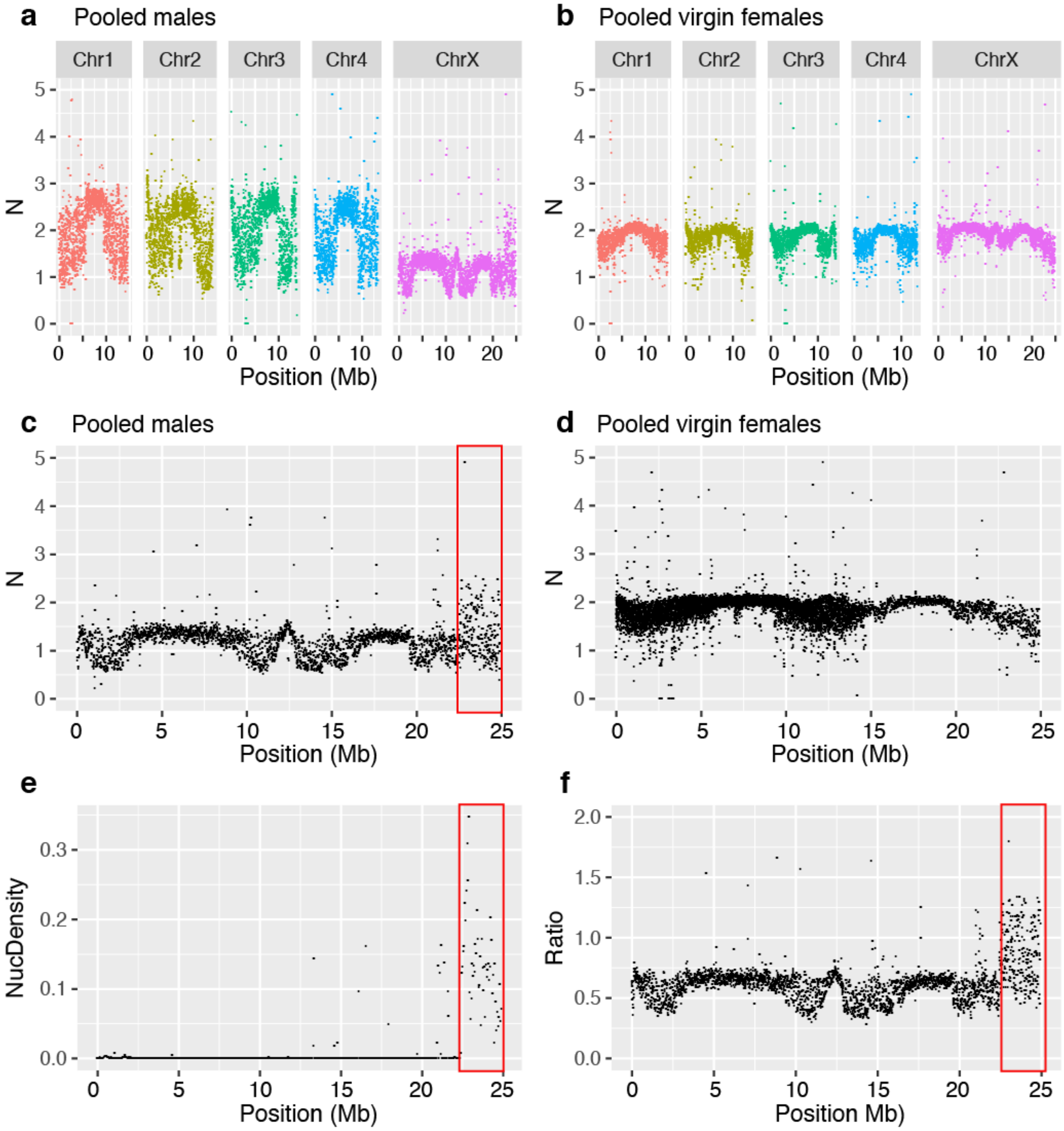

Supplementary Figure 2. Pseudoautosomal region of *B. malayi*
The normalized sequencing depth (N) is plotted for (**a**) pooled data from 22 males for each position in all chromosomes; (**b**) a pool of virgin females for each position in all chromosomes; (**c**) pooled data from 22 males for each position in the X chromosome; (**d**) a pool of virgin females for each position in the X chromosome. (**e**) The density of heterozygous SNPs (Pi) in 10 kb windows from the male data is plotted across the X chromosome. (**f**) The ratio of the normalized sequencing depth between females and males is plotted across the X chromosome. Red box highlights PAR region on X chromosome.

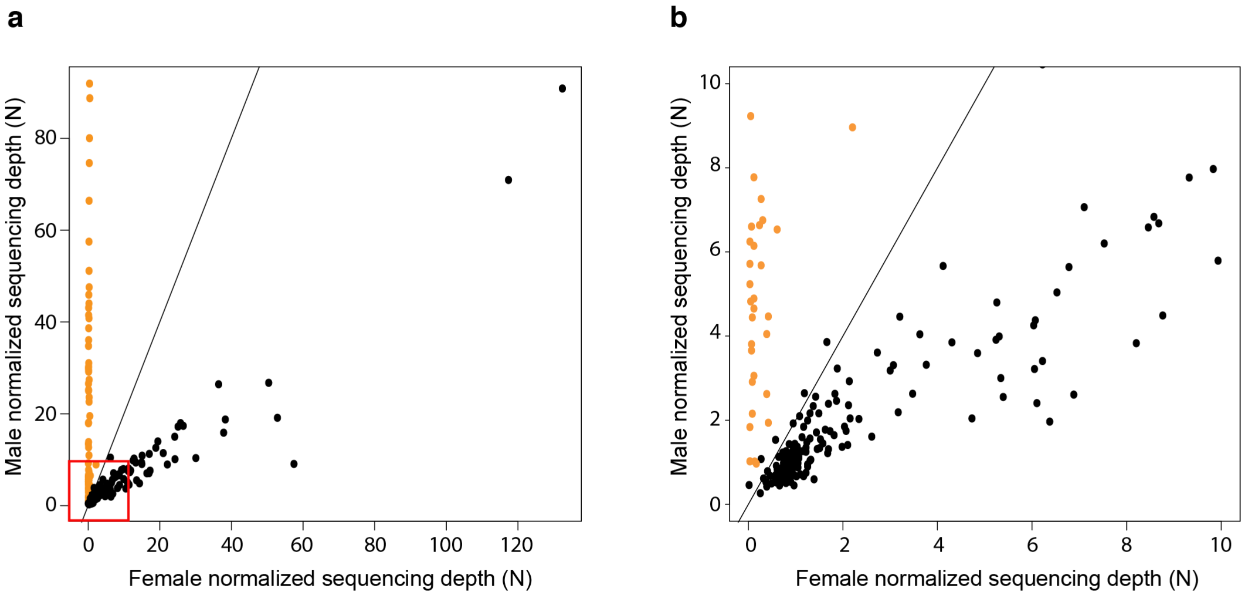

Supplementary Figure 3. Sequencing depth from virgin females compared to males normalized to the average of each sample

The average sequencing depth calculated for each contig is compared between a pool of virgin females and pooled data from individual males with putative chromosome Y-specific contigs are highlighted in orange for (**a**) all data and (**b**) a subset of data where both points are <10N. The line represents where the male sequencing depth is twice the virgin female sequencing depth.

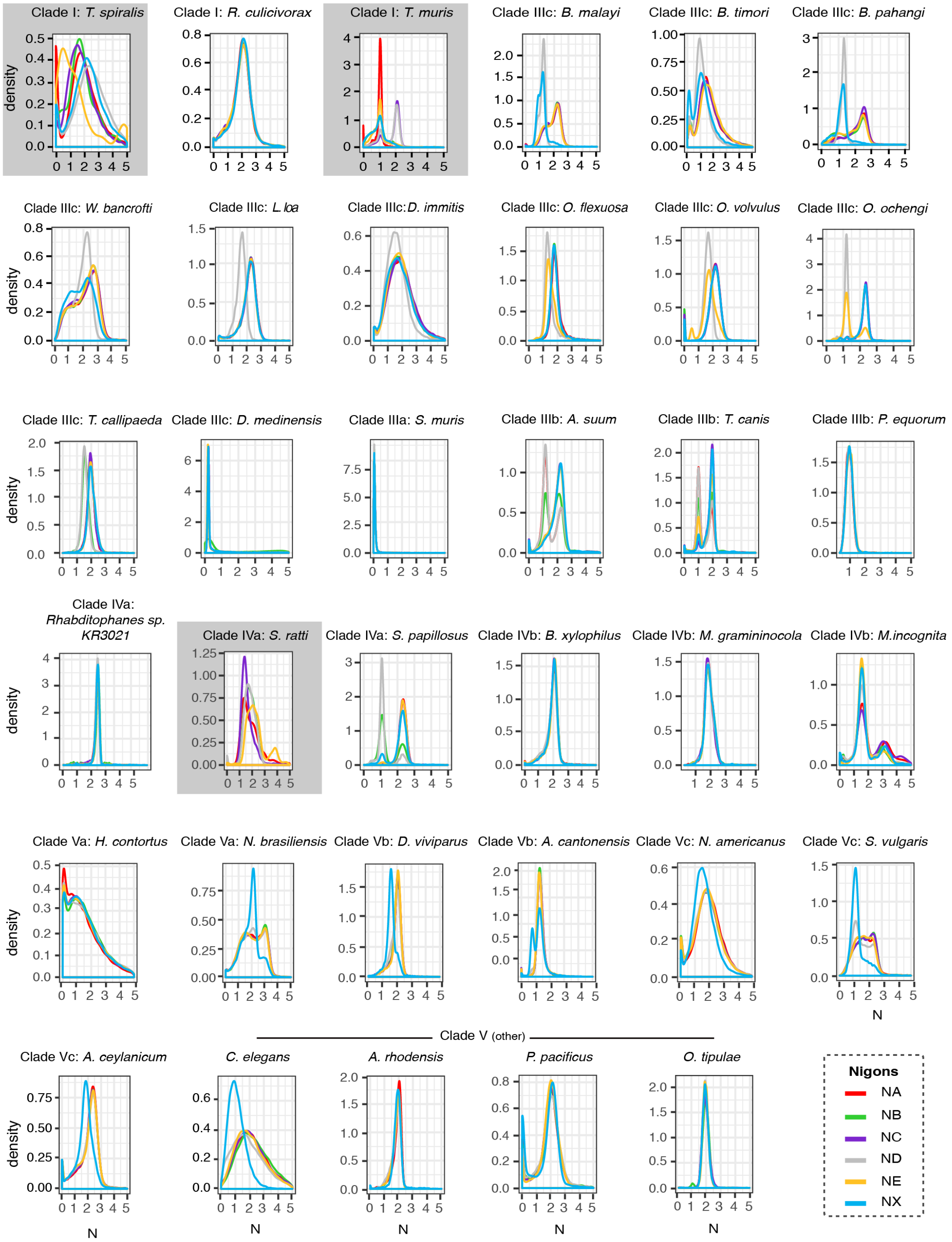

Supplementary Figure 4. Density plot of the sequencing depth over Nigon elements for species across the phylum Nematoda

The density of depth per Nigon element was calculated in R for positions and aggregated by the predicted Nigon elements. The modes for these plots were calculated for all values of N > 0.2 and presented in **Supplementary Data 2**. *B. malayi*, *B. pahangi* and *W. bancrofti* all share similar unpaired chromosome segments in males corresponding to ND and NX. *L. loa* appears to only be unpaired in males for ND, while all three *Onchocerca* species are unpaired in males for ND and NE. *D. viviparus* and *N. americanus* both appear to be unpaired in males for NX only. The grey boxes around *S. ratti*, *T. spiralis*, and *T. muris* highlight the limitations of the analysis method for complete genomes with fused chromosomes, as described in the methods.

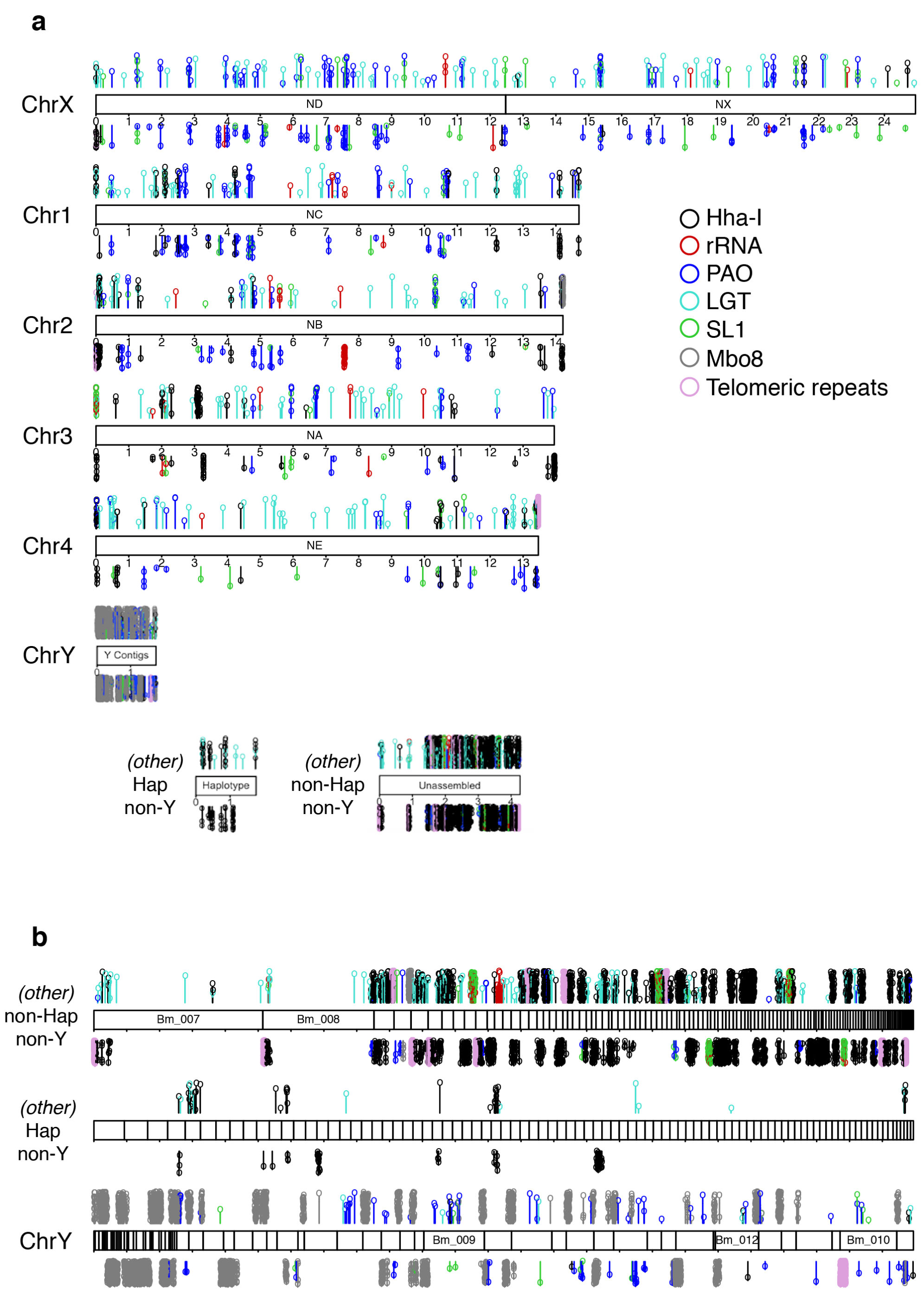

Supplementary Figure 5. Repeat distribution across chromosomes

(a) Repeat distribution of major families across the *B. malayi* genome. Hidden Markov models were generated for the HhaI repeats (black), MBO8 repeats (gray), and telomeric repeats (plum) which were then applied to the *B. malayi* genome in order to determine their genomic locations. These were plotted in conjunction with the rRNA (red), PAO repeats (blue), SL1 (green) and lateral gene transfers – LGT (turquoise) across the genome. Pseudo-chromosomes were formed using non-chromosomal contigs. All contigs that were identified as Y specific form a 1.7 Mb pseudo Y chromosome; the non-Y non-haplotype contigs form a 4.3 Mb pseudo-contig, and the predicted 1.7 Mb non-Y haplotype contigs form a third pseudo-contig. **(b)** Zoomed-in view of the 3 pseudo-chromosomes/contigs.

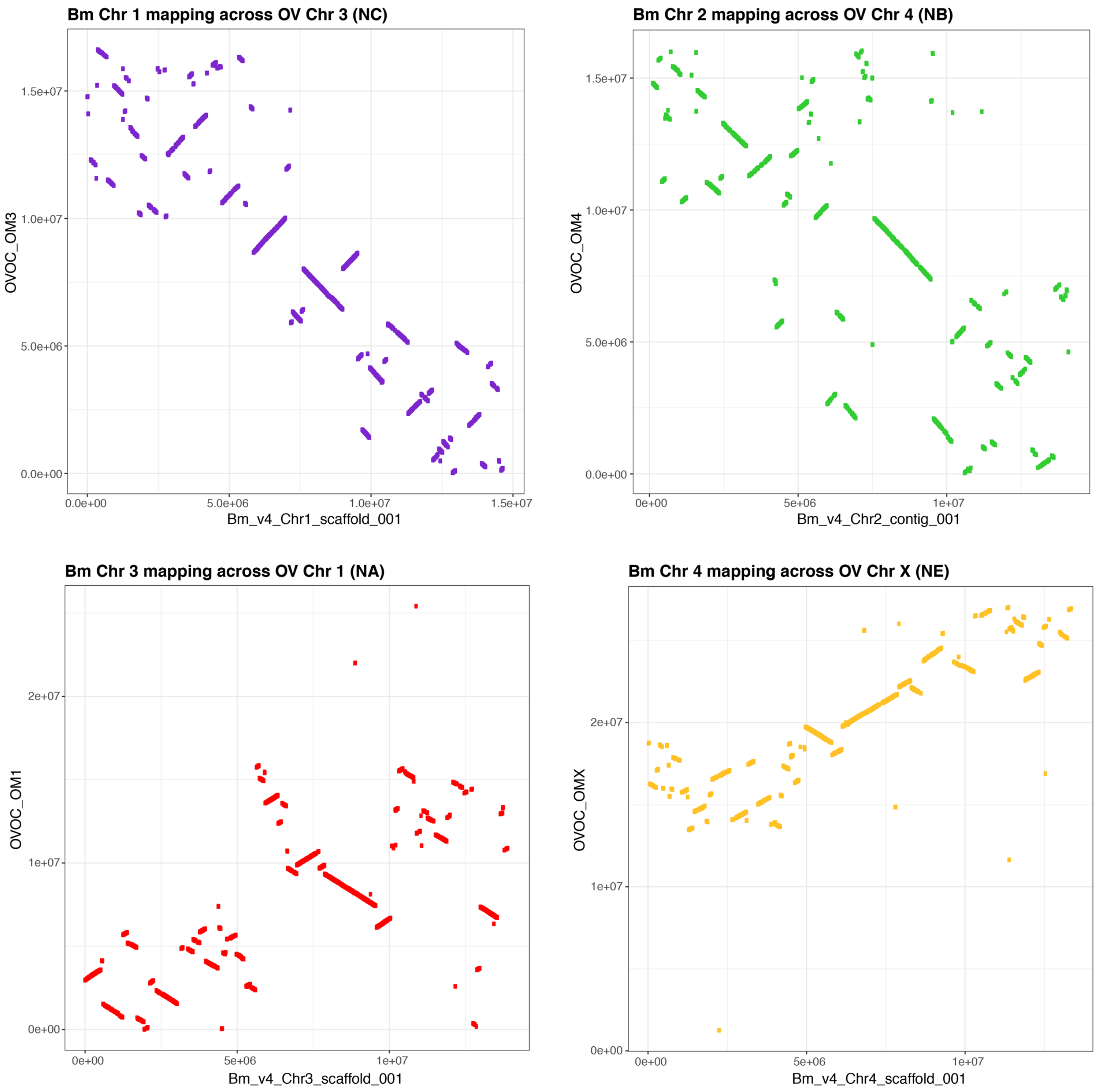

Supplementary Figure 6. ***B. malayi*** **autosome mapping across** ***O. volvulus*** **chromosomes**
The 4 panels show the synteny of the *B. malayi* chromosomes (autosomes) to corresponding chromosomes and Nigon elements in *O. volvulus.* The axes provide chromosome or scaffold locations in Mb.

Supplementary Table 1. Genome properties

	B. malayi v3.0	***B. malayi v4.0***
Assembly version	WS242	**WS267**
Assembly size (Mb)	94	**88**
♯ scaffolds[a]	9,827	**197**
N50 of scaffolds (Mb)	0.191	**14.2**
N50 (num)	62	**3**
N90 of scaffolds (Mb)	0.002	**13.5**
N90 (num)	2,451	**5**
Maximum length of scaffold (Mb)	5.2	**24.9**
G+C content (%)	27	**28**
Sequence coverage (% not gap)	83.5	**99.37**
[b]Cegma completeness (%): (complete/partial)	96.77/97.18	**97.18/97.58**
No. of protein coding genes	14,114	**11,018**
Gene density (genes/Mb)	150	**125**
Mean protein aa length	370	**479**
Median protein aa length	241	**349**
No. coding exons	137,127	**135,429**
Coding exons, combined length (Mb)	19.8	**20.2**
Mean no. of coding exons per gene	9.8	**12**
Mean coding exon length (bp)	145	**149**
Median coding exon length (bp)	133	**135**
No. of introns	121,035	**128,347**
Mean intron length (bp)	337	**349**
Median intron length (bp)	223	**226**

[a]Scaffold number does not include haplotypes.
[b]Assembly completeness was estimated by CEGs (Core Eukaryotic Genes) with the CEGMA v2 software. The 4 missing genes in *B. malayi* v4.0 correspond to KOGs 1468,2303, 2531 and 2770 and are missing in other filarial genomes.

Supplementary Table 2. Selection process of upregulated genes used for motif discovery in *Brugia malayi*

RNA-seq experiment	Sex	Total number of up-regulated genes	Number of up-regulated genes used to perform motif discovery	Number of motifs by ensemble motif discovery	Number of motifs after *in silico* evaluation	Number of matches in motif databases	Number of conserved motifs
**30 dpi**	Male	566	205 (cutoff at |logFC|>=3)	2847	41	11	11
	Female	473	132 (cutoff at |logFC|>=3)	2820	41	6	4
**120 dpi**	Make	2375	174 (cutoff at |logFC|>=6)	2801	38	5	2
	Female	2032	188 (cutoff at |logFC|>=5)	2818	64	12	12
