# Supplementary material for: Sex chromosome evolution in parasitic nematodes of humans
Source: Nat Commun. 2020 Apr 23;11:1964. doi: 10.1038/s41467-020-15654-6 (PMC7181701; doi:10.1038/s41467-020-15654-6)
Supplement: Supplementary file 2 — reporting summary [file 41467_2020_15654_MOESM2_ESM.pdf]

natureresearch

Corresponding author(s): Dr. Elodie Ghedin

Last updated by author(s): Mar 10, 2020



# Reporting Summary

Nature Research wishes to improve the reproducibility of the work that we publish. This form provides structure for consistency and transparency in reporting. For further information on Nature Research policies, see Authors & Referees and the Editorial Policy Checklist .

## Statistics

For all statistical analyses, confirm that the following items are present in the figure legend, table legend, main text, or Methods section.

| n/a | Confirmed | |
|---|---|---|
| ☒ | ☐ | The exact sample size (*n*) for each experimental group/condition, given as a discrete number and unit of measurement |
| ☐ | ☒ | A statement on whether measurements were taken from distinct samples or whether the same sample was measured repeatedly |
| ☐ | ☒ | The statistical test(s) used AND whether they are one- or two-sided<br>*Only common tests should be described solely by name; describe more complex techniques in the Methods section.* |
| ☒ | ☐ | A description of all covariates tested |
| ☒ | ☐ | A description of any assumptions or corrections, such as tests of normality and adjustment for multiple comparisons |
| ☒ | ☐ | A full description of the statistical parameters including central tendency (e.g. means) or other basic estimates (e.g. regression coefficient) AND variation (e.g. standard deviation) or associated estimates of uncertainty (e.g. confidence intervals) |
| ☒ | ☐ | For null hypothesis testing, the test statistic (e.g. *F*, *t*, *r*) with confidence intervals, effect sizes, degrees of freedom and *P* value noted<br>*Give P values as exact values whenever suitable.* |
| ☒ | ☐ | For Bayesian analysis, information on the choice of priors and Markov chain Monte Carlo settings |
| ☒ | ☐ | For hierarchical and complex designs, identification of the appropriate level for tests and full reporting of outcomes |
| ☒ | ☐ | Estimates of effect sizes (e.g. Cohen's *d*, Pearson's *r*), indicating how they were calculated |

*Our web collection on statistics for biologists contains articles on many of the points above.*

## Software and code

Policy information about availability of computer code

| | |
|---|---|
| Data collection | Argus Optical Mapping System and associated MapManager (2015) and MapSolver (v3.1) |
| Data analysis | edgeR v3.26.8, SMALT v0.5.2, PhyML v3.1, GimmeMotifs v0.10.0b6, DME v2.0, DECODE v1.01, gkm-SVM 1.3, HOMER 2.0, AMD 1.0, BioProspector 1.0, MDmodule 1.0, MEME 4.11.2, Weeder 2.0, GADEM v1.3, Improbizer 1.0, TOMTOM 4.11.2, Infernal 1.1rc1, OpGen's GenomeBuilder (2015), HGAP 2.0, gap5 v1.2.14, ICORN2, Bowtie v2.2.3, Image (no versions - https://sourceforge.net/projects/image2/), PBJelly and Quiver from PBSuite_14.6.24 and smrtanalysis-2.2.0.133377 Sprai-0.9.9.1, CEGMA v2, promer v3.23, mummer v3.23, R v3.4.4, Circos v0.69-3, mummerplot v.3.23, BWA v0.7.12, SAMtools v1.1, NCBI BLAST v.2.8.1, PRAZE (does not have a version), Exonerate v2.2.0, Augustus v3.1, Nucmer v.3.23, Picard Tools v2.5.0, ggplot2 v3.1.0., RepeatModeller v1.0.8, RepeatMasker v4.0.3, MUSCLE v3.8.31, mySQL v5.1, GenBlastG v139, Infernal v1.1rc1, ATAC (2008), SignalP v4, Cufflinks v2.2.1, NCBI BLAST v.2.8.1 |

For manuscripts utilizing custom algorithms or software that are central to the research but not yet described in published literature, software must be made available to editors/reviewers. We strongly encourage code deposition in a community repository (e.g. GitHub). See the Nature Research guidelines for submitting code & software for further information.

## Data

Policy information about availability of data

All manuscripts must include a data availability statement. This statement should provide the following information, where applicable:

- Accession codes, unique identifiers, or web links for publicly available datasets
- A list of figures that have associated raw data
- A description of any restrictions on data availability

B. malayi v4 assembly with the WS270 annotation are available in the European Nucleotide Archive (ENA) database under accession number GCA_000002995.5 [https://www.ebi.ac.uk/ena/data/view/GCA_000002995.5], as well as at WormBase [http://www.wormbase.org/species/b_malayi] and WormBase-Para-Site

[http://parasite.wormbase.org/Brugia_malayi_prjna10729/Info/Index/]. Illumina HiSeq 2000 paired end sequencing data from virgin female resequencing data are available at ERS992391 [http://www.ebi.ac.uk/ena/data/view/ERS992391]. The PacBio data is available under PRJNA421950 [https://www.ncbi.nlm.nih.gov/bioproject/PRJNA421950]. Spliced leader RNAseq reads are available under PRJNA525735 [https://www.ncbi.nlm.nih.gov/bioproject/PRJNA525735]. Accession numbers for each dataset obtained from public data, and used in the analyses, are listed in Supplementary Data 2.

# Field-specific reporting

Please select the one below that is the best fit for your research. If you are not sure, read the appropriate sections before making your selection.

☒ Life sciences ☐ Behavioural & social sciences ☐ Ecological, evolutionary & environmental sciences

For a reference copy of the document with all sections, see nature.com/documents/nr-reporting-summary-flat.pdf

# Life sciences study design

All studies must disclose on these points even when the disclosure is negative.

| | |
|---|---|
| Sample size | Sample size was not a consideration in the design. |
| Data exclusions | No data was excluded. |
| Replication | Replicates were used in the analysis of RNASeq data. In addition, replicate individual males were analyzed. |
| Randomization | No randomization was used in this study. |
| Blinding | No blinding was used in this study. |

# Reporting for specific materials, systems and methods

We require information from authors about some types of materials, experimental systems and methods used in many studies. Here, indicate whether each material, system or method listed is relevant to your study. If you are not sure if a list item applies to your research, read the appropriate section before selecting a response.

## Materials & experimental systems

| n/a | Involved in the study | |
|---|---|---|
| ☒ | ☐ | Antibodies |
| ☒ | ☐ | Eukaryotic cell lines |
| ☒ | ☐ | Palaeontology |
| ☐ | ☒ | Animals and other organisms |
| ☒ | ☐ | Human research participants |
| ☒ | ☐ | Clinical data |

## Methods

| n/a | Involved in the study | |
|---|---|---|
| ☒ | ☐ | ChIP-seq |
| ☒ | ☐ | Flow cytometry |
| ☒ | ☐ | MRI-based neuroimaging |

## Animals and other organisms

Policy information about studies involving animals; ARRIVE guidelines recommended for reporting animal research

| | |
|---|---|
| Laboratory animals | All B. malayi parasite material was obtained from intraperitonEal infections of gerbils (Meriones unguiculatus) maintained by TRS Labs (Athens, GA, USA) or by the FR3 (Filariasis Research Reagent Resource Center; BEI Resources, Manassas, VA, USA). The FR3 and TRS life cycles have been maintained independently for decades, but were initiated with material from the same clinical infection Live adult parasites were shipped from TRS Labs to New England Biolabs (NEB) for preparation of DNA for sequencing on the Pacific Biosciences single molecule real-time sequencing platform and for optical mapping.<br><br>Infected gerbils were shipped from the FR3 to the University of Wisconsin, Oshkosh for recovery of virgin female worms. Animal work was conducted under the University Institutional Animal Care and Use protocol (#0026-000246-R1-09-14). |
| Wild animals | No wild animals were used |
| Field-collected samples | No field-collected samples were used in this study |
| Ethics oversight | Animal work was conducted under the University Institutional Animal Care and Use protocol (#0026-000246-R1-09-14). |

Note that full information on the approval of the study protocol must also be provided in the manuscript.
