# Supplementary material for: Sex chromosome evolution in parasitic nematodes of humans
Source: Nat Commun. 2020 Apr 23;11:1964. doi: 10.1038/s41467-020-15654-6 (PMC7181701; doi:10.1038/s41467-020-15654-6)
Supplement: Supplementary file 3 — description of Additional Supplementary Files [file 41467_2020_15654_MOESM3_ESM.docx]

Description of Additional Supplementary Files

**File name: Supplementary Data 1**

Description: Sequencing Depth and calculations of N for the single males (paired end), the pool of gravid *Wolbachia*-depleted females (mate pair and paired end), and the pool of the virgin females (paired end) that were used for determining the putative Y contigs

**File name: Supplementary Data 2**

Description: Sequencing depth modes for each Nigon element in nematode species analyzed

**File name: Supplementary Data 3**

Description: Genes found conserved on X chromosomes of *C. elegans*, *O. volvulus*, and *B. malayi*

**File name: Supplementary Data 4**

Description: Distribution of repeats by Nigon element

**File name: Supplementary Data 5**

Description: Location of integrated SL1s associated with PAO retrotransposons

**File name: Supplementary Data 6**

Description: Characteristics, distribution, and analysis of nuwts and numts

**File name: Supplementary Data 7**

Description: *B. malayi* genes with sex-biased gene expression in males and females at 30 or 120 days post-infection (dpi)

**File name: Supplementary Data 8**

Description: Sex-biased motifs enriched in worms at 30 days post-infection (dpi)

**File name: Supplementary Data 9**

Description: Sex-biased motifs enriched in worms at 120 days post-infection (dpi)

**File name: Supplementary Data 10**

Description: Y contig genes of *B. malayi* or *O. volvulus* and *C. elegans* orthologs
